# Thyroid Hyperplasia and Neoplasm Adverse Events Associated With Glucagon-Like Peptide-1 Receptor Agonists in the Food and Drug Administration Adverse Event Reporting System: Retrospective Analysis

**DOI:** 10.2196/55976

**Published:** 2024-05-01

**Authors:** Tigran Makunts, Haroutyun Joulfayan, Ruben Abagyan

**Affiliations:** 1 Skaggs School of Pharmacy and Pharmaceutical Sciences, University of California San Diego, La Jolla, CA, United States; 2 University of California San Diego, La Jolla, CA, United States

**Keywords:** GLP-1, FDA, averse event reporting, cancer, oncology, neoplasm, drugs, pharmacy, pharmacology, pharmaceutics, medication, medications, glucagon-like peptide-1, Food and Drug Administration, weight loss, diabetes, obesity, thyroid hyperplasia, FAERS, FDA Adverse Event Reporting System

## Abstract

**Background:**

Glucagon-like peptide-1 (GLP-1) receptor agonists (RAs) are one of the most commonly used drugs for type 2 diabetes mellitus. Clinical guidelines recommend GLP-1 RAs as an adjunct to diabetes therapy in patients with chronic kidney disease, presence or risk of atherosclerotic cardiovascular disease, and obesity. The weight loss observed in clinical trials has been explored further in healthy individuals, putting GLP-1 RAs on track to be the next weight loss treatment.

**Objective:**

Although the adverse event profile is relatively safe, most GLP-1 RAs come with a labeled boxed warning for the risk of thyroid cancers, based on animal models and some postmarketing case reports in humans. Considering the increasing popularity of this drug class and its expansion into a new popular indication, a further review of the most recent postmarketing safety data was warranted to quantify thyroid hyperplasia and neoplasm instances.

**Methods:**

GLP-1 RA patient reports from the US Food and Drug Administration (FDA) Adverse Event Reporting System database were analyzed using reporting odds ratios and 95% CIs.

**Results:**

In this study, we analyzed over 18 million reports from the US FDA Adverse Event Reporting System and provided evidence of significantly increased propensity for thyroid hyperplasias and neoplasms in patients taking GLP-1 RA monotherapy when compared to patients taking sodium-glucose cotransporter-2 (SGLT-2) inhibitor monotherapy.

**Conclusions:**

GLP-1 RAs, regardless of indication, are associated with an over 10-fold increase in thyroid neoplasm and hyperplasia adverse event reporting when compared to SGLT-2 inhibitors.

## Introduction

Glucagon-like peptide-1 (GLP-1) receptor agonists (RAs) have gained increased popularity due to the improved safety and efficacy profiles observed in clinical trials. This class of drugs includes liraglutide, semaglutide, exenatide, and dulaglutide [1-5]. GLP-1 RAs are indicated as a therapeutic adjunct to diet and exercise, to improve glycemic control in patients with type 2 diabetes mellitus (T2DM). The American Association of Clinical Endocrinology and the American Diabetes Association recommend GLP-1 RAs for patients with T2DM who have chronic kidney disease, atherosclerotic cardiovascular disease risk, or obesity [6,7]. GLP-1 RAs have also been used in a wide range of cardiometabolic conditions, including but not limited to nonalcoholic fatty liver disease and nonalcoholic steatohepatitis [8].

GLP-1 RAs were recommended for patients with both T2DM and obesity because of the weight loss observed during the clinical trials [2-5,9]. The effect was attributed to decreasing gastric emptying, peristalsis, appetite, and glucose absorption [10,11]. This effect was further explored in studies of populations without T2DM [12-14], suggesting the expansion of GLP-1 RA use.

Common adverse events (AEs) associated with GLP-1 RAs observed during the clinical trials include nausea, hypoglycemia, vomiting, diarrhea, feeling jittery, dizziness, headache, and dyspepsia. Of a greater concern are the labeled boxed warnings of liraglutide, semaglutide, and dulaglutide, marking these as contraindicated in patients with a family history of medullary thyroid carcinoma. These warnings were based on nonhuman data about the development of thyroid C-cell tumors in rats and mice receiving clinically relevant doses of GLP-1 RAs [15-17]. The thyroid cancer association in humans has been studied and observed as well in retrospective studies [18-20]. However, there is conflicting evidence from a meta-analysis of human randomized controlled trials, which refutes this association [21].

The increased popularity of GLP-1 RAs and the unsettled association of thyroid hyperplasias and neoplasms prompted further investigation into the most recent US Food and Drug Administration (FDA) Adverse Event Reporting System (FAERS) data sets. In this study, we evaluated thyroid hyperplasia and neoplasm–related AEs that are reported as being associated with GLP-1 RA monotherapy when compared to sodium-glucose cotransporter-2 (SGLT-2) inhibitors monotherapy. SGLT-2 inhibitors, a class of drugs that work by blocking renal glucose reabsorption, were selected as the control cohort due to their comparable indication in diabetes treatment guidelines. We analyzed GLP-1 RAs individually and as a class.

## Methods

### Ethical Considerations

Ethical review and approval were not required for the study on human participants in accordance with local legislation and institutional requirements. Written informed consent from the participants’ legal guardian or next of kin was not required to participate in this study in accordance with national legislation and institutional requirements. The data used for the analysis had been deidentified and made public by the US FDA.

### FAERS Data Sets

The FAERS is a repository of AE cases sent to the FDA through MedWatch (form 3500/3500a) [22,23]. The cases include AEs submitted voluntarily by health care professionals, individuals, and legal representatives and spontaneous mandatory reports by manufacturers and sponsors. At the time of the analysis, the FAERS contained 18,274,795 reports from January 2004 to September 2022.

The data sets have been deidentified and made available on the web [24].

### Data Preparation, Cohort Selection, and Outcome Measure

The FAERS quarterly data sets were initially downloaded in text format. Due to the variability of data structure between quarters and the paucity of some of the variables, the cases were standardized to fit a uniform structure [25-27].

Out of a total of 18,274,795 reports, AE cases where the report was submitted by a health care professional (pharmacists, physicians, nurses, or other health care professionals) were selected as the initial data set (n=6,360,489). This allowed for minimizing reporting bias and adding to the clinical relevance of the studied cases. Further, reports where GLP-1 RAs were the only drug reported (further referred to as *monotherapy*) were selected as the GLP-1 RA cohort (n=17,653) to avoid potential confounding effects from concomitant medications. The cohort was further split into individual GLP-1 RA subcohorts: semaglutide (n=3230), dulaglutide (n=3768), exenatide (n=4493), and liraglutide (n=6162). SGLT-2 inhibitor monotherapy reports (canagliflozin, dapagliflozin, and empagliflozin; n=14,102) were selected as a control cohort. SGLT-2 inhibitors were selected as a control due to the comparable recommendation by diabetes management guidelines. Metformin monotherapy was initially selected as the control cohort to match the population of the cohort of interest (n=8536); however, only a single case of interest (Preferred Term [PT] code: thyroid cancer) was reported. The undetectable baseline of this AE made it impossible to use metformin as a control (Table 1). All the thyroid-related AE PT codes based on standard MedDRA queries and FDA Medical queries were used in the case selection process. Disproportionality analysis using reporting odds ratios (RORs) and 95% CIs was used to determine the statistical significance of the results. Of the 31,755 included reports, only those from health care professionals (pharmacists: n=3620, 11.4%; physicians: n=20,133, 63.4%; and other health care professionals: n=8002, 25.2%) were included in the analysis. The reports were primarily from the United States (United States: n=26,452, 83.3%; Japan: n=603, 1.9%; United Kingdom: n=540, 1.7%; France: n=476, 1.5%; Canada: n=349, 1.1%; Australia: n=349, 1.1%; and other counties: n=2986, 9.4%, <1% in each country). The reports were from the following years: 2010 (n=2128, 6.7%), 2011 (n=3430, 10.8%), 2012 (n=2096, 6.6%), 2013 (n=1143, 3.6%), 2014 (n=1080, 3.4%), 2015 (n=1969, 6.2%), 2016 (n=1429, 4.5%), 2017 (n=1651, 5.2%), 2018 (n=2159, 6.8%), 2019 (n=2191, 6.9%), 2020 (n=4096, 12.9%), 2021 (n=4954, 15.6%), and 2022 (n=3430, 10.8%; only the first 3 quarters at the time of the analysis).

**Table 1.** Thyroid hyperplasia or neoplasm–related AE^a^ reports in GLP-1^b^ RA^c^, metformin, and SGLT-2^d^ inhibitor FAERS^e^ reports.

AE Preferred Term	AE reports, n (%)					
	Semaglutide (n=3230)	Dulaglutide (n=3768)	Exenatide (n=4493)	Liraglutide (n=6162)	Metformin (n=8536)	SGLT-2 inhibitors (canagliflozin, empagliflozin, and dapagliflozin; control^f^; n=14,102)
Thyroid mass	19 (0.59)	9 (0.24)	5 (0.11)	25 (0.41)	0 (0)	1 (0.01)
Medullary thyroid cancer	8 (0.25)	5 (0.13)	1 (0.02)	7 (0.11)	0 (0)	2 (0.01)
Thyroid cancer	3 (0.09)	10 (0.27)	4 (0.09)	30 (0.49)	1 (0.01)	3 (0.02)
Papillary thyroid cancer	2 (0.06)	3 (0.08)	2 (0.04)	20 (0.32)	0 (0)	0 (0)
Benign neoplasm of thyroid gland	2 (0.06)	2 (0.05)	2 (0.04)	1 (0.02)	0 (0)	1 (0.01)
Thyroid neoplasm	0 (0)	2 (0.05)	3 (0.07)	13 (0.21)	0 (0)	1 (0.01)
Thyroid cyst	0 (0)	2 (0.05)	0 (0)	6 (0.10)	0 (0)	0 (0)
Follicular thyroid cancer	0 (0)	0 (0)	1 (0.02)	1 (0.02)	0 (0)	0 (0)
Thyroid adenoma	0 (0)	0 (0)	0 (0)	4 (0.06)	0 (0)	0 (0)
Thyroid C-cell hyperplasia	0 (0)	0 (0)	0 (0)	1 (0.02)	0 (0)	0 (0)
Thyroid cancer metastatic	0 (0)	0 (0)	0 (0)	2 (0.03)	0 (0)	0 (0)
*Unique individual thyroid hyperplasia or neoplasm–related AE reports^g^*	*33 (1.02)*	*33 (0.88)*	*17 (0.38)*	*108 (1.75)*	*1 (0.01)*	*7 (0.05)*

a AE: adverse event.

b GLP-1: glucagon-like peptide-1.

c RA: receptor agonist.

d SGLT-2: sodium-glucose cotransporter-2.

e FAERS: Food and Drug Administration Adverse Event Reporting System.

f Control cohort.

g The total number of unique individual reports was used for the analysis to avoid overcounting cases that had more than 1 AE of interest listed.

### Statistical Analysis

#### Descriptive Statistics

Frequencies for each AE PT code were calculated by the following equation:


$$Frequency\;\left(\%\right)=\frac{\left(Number\;of\;reports\;with\;AE\;in\;a\;cohort\right)\times \;100}{Number\;of\;reports\;in\;a\;cohort}$$

#### Comparative Statistics

AE report rates were compared via the ROR analysis using the following equations:


$$ROR=\frac{\left(a\;÷\;b\right)}{\left(c\;÷\;d\right)}$$

LnROR=Ln(ROR)

$$SE_{LnROR}=\sqrt{1/a+1/b+1/c+1/d}$$

$$\begin{array}{c} 95\%\;CI=\left[exp\left(LnROR-1.96\times SE_{LnROR}\right),\;exp\left(LnROR+1.96\times SE_{LnROR}\right)\right] \end{array}$$

where a is the number of AE cases in the exposed group, b is the number of non-AE cases in the exposed group, c is the number of AE cases in the control group, and d is the number of non-AE cases in the control group.

## Results

A total of 31,755 monotherapy reports, including 17,653 GLP-1 RA and 14,102 SGLT-2 inhibitor reports, were used for the analysis of 191 unique thyroid hyperplasia or neoplasm reports in the GLP-1 RA group and 7 reports in the SGLT-2 group, respectively (Tables1  2).

**Figure 1.**
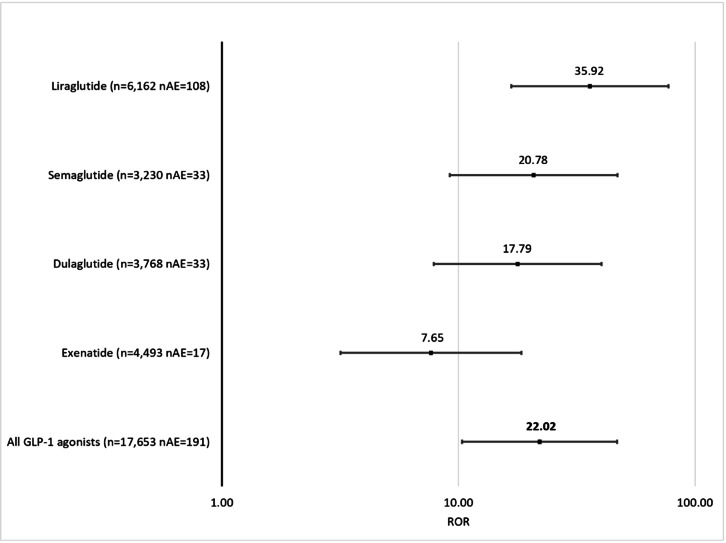
Reporting odds ratios (RORs) of thyroid hyperplasia or neoplasm–related AEs in individual GLP-1 RA monotherapy cohorts and GLP-1 RAs as a class, when compared to the SGLT-2 inhibitor monotherapy control cohort. The x-axis is presented in logarithmic scale. AE: adverse event; GLP-1: glucagon-like peptide-1; nAE: number of adverse events; RA: receptor agonist; SGLT-2: sodium-glucose cotransporter-2.

GLP-1 RA monotherapy reports showed a statistically significant increase in thyroid hyperplasia and neoplasm AEs, with the ROR being 22.02 (95% CI 10.36-46.84) when compared to SGLT-2 inhibitors. When analyzed individually, every GLP-1 RA had significantly increased reporting of thyroid hyperplasia or neoplasm–related AEs when compared to SGLT-2 inhibitors. The results for all the GLP-1 RAs showed that the lower bound of the 95% CI of the ROR range was above 3: semaglutide (ROR 20.78, 95% CI 9.19-47.03), dulaglutide (ROR 17.79, 95% CI 7.86-40.25), exenatide (ROR 7.65, 95% CI 3.17-18.45), and liraglutide (ROR 35.92, 95% CI 16.71-77.20; Figure 1).

**Table 2.** Number of AE^a^ reports and the respective demographics.

Demographics		Drug cohort				
		Semaglutide (n=3230)	Dulaglutide (n=3768)	Exenatide (n=4493)	Liraglutide (n=6162)	SGLT-2^b^ inhibitors (canagliflozin, empagliflozin, and dapagliflozin; control^c^; n=14,102)
Unique individual thyroid hyperplasia or neoplasm reports, n (%)		33 (1.02)	33 (0.88)	17 (0.38)	108 (1.75)	7 (0.05)
**Sex, n (%)**						
	Male	4 (0.12)	14 (0.37)	3 (0.07)	35 (0.57)	3 (0.02)
	Female	27 (0.84)	14 (0.37)	13 (0.29)	66 (1.07)	2 (0.01)
	Unknown	2 (0.06)	5 (0.13)	1 (0.02)	7 (0.11)	2 (0.01)
**Age (y)**						
	Median	56	60	61	55	N/A^d^
	Mean (SD)	55.94 (13.41)	57.33 (11.35)	64.67 (6.35)	53.07 (11.08)	N/A

a AE: adverse event.

b SGLT-2: sodium-glucose cotransporter-2.

c Control cohort.

d N/A: not applicable.

## Discussion

### Principal Findings

In this study, we observed a reported association of GLP-1 RA treatment with thyroid hyperplasia and neoplasm AEs. To our knowledge, this is the first analysis of the FAERS to generate an ROR profile of GLP-1 RA monotherapy as a class and further analyze the individual GLP-1 RA monotherapy AEs compared to SGLT-2 inhibitors monotherapy AEs. The mean ages of the specific drug cohorts with these AEs were similar for comparison, ranging from 53.0 to 64.7 years. The thyroid hyperplasia and neoplasm AE association was significant for every GLP-1 RA in the class, with an even narrower range of 95% CI in the combined cohort. The RORs ranged from 7.65 to 35.92, with the lowest bound being 3.17. The highest mean ROR value was observed for liraglutide, and the lowest mean ROR was observed for exenatide, with only a small overlap in the 95% CIs. Interestingly, this trend was also seen in a similar analysis performed by Mali et al [20] using the European pharmacovigilance database (EudraVigilance), where this association was the strongest in the liraglutide cohort, followed by the exenatide cohort, with proportional reporting ranges of 27.5 (95% CI 22.7-33.3) and 22.5 (95% CI 17.9-28.3), respectively. The differences in the numbers and 95% CI ranges are due to the numbers of reports and type of analysis.

An association between T2DM and thyroid function has been previously established [28]. These disease states are often comorbid, and one affects the disease progression of the other [29]. However, the potential molecular mechanisms responsible for thyroid effects of GLP-1 RAs are insufficiently characterized and may include multiple pathways such as phosphoinositol-3 kinase and Akt serine/threonine kinase pathways; mitogen-activated protein kinase and extracellular signal-regulated kinase pathways; expression of GLP-1 receptors by C-cells; and GLP-1 association with triiodothyronine levels [21,30]. Thyroid hormone plays a pivotal role in nearly every aspect of lipid metabolism [31,32]. Thus, it was expected to observe thyroid hyperplasia and neoplasm–related AEs across all the T2DM drug cohorts that were investigated. However, only a single report of thyroid hyperplasia and neoplasm AE (thyroid cancer) was observed in the metformin monotherapy cohort, and only 7 were observed in the SGLT-2 inhibitor monotherapy cohort that was selected as the control due to similar T2DM disease-stage treatment guideline recommendations. In contrast, almost 200 of these AEs were reported for GLP-1 RAs in similarly sized cohorts, resulting in a statistically significant reported association. Therefore, as the GLP-1 RAs expand into non-T2DM indications such as obesity metabolic syndrome and other related conditions, controlled studies and better understanding of the molecular mechanisms of action are necessary to investigate this association and prevent potential serious consequences.

### Study Limitations

Since this is an association study, the causality between the thyroid hyperplasias and neoplasms and GLP-1 RAs was not clinically adjudicated. However, this analysis of a large population–scale AE database provides a strong signal that may be clinically significant. The numbers of AEs presented in the study do not represent all treated patients due to voluntary submissions resulting in over- and underreporting [33,34]. Additionally, due the nature and long progression of the studied AEs, often requiring invasive procedures for a proper diagnosis, the cases may be significantly underreported. The case narratives with additional information such as a thorough medical history and test or diagnostic results were not available in the data sets provided by the FDA due to privacy concerns. Other limitations include the possible over-the-counter medications and supplements that are often not reported to health care professionals and may potentially add noise to the cohort compositions, AE frequencies, and to a lesser extent, RORs and the respective CIs. However, considering the large number of individuals in the GLP-1 RA and control cohorts, which were matched by indication, the signal was statistically significant.

## Abbreviations

AE: adverse event
FAERS: Food and Drug Administration Adverse Event Reporting System
FDA: Food and Drug Administration
GLP-1: glucagon-like peptide-1
PT: Preferred Term
RA: receptor agonist
ROR: reporting odds ratio
SGLT-2: sodium-glucose cotransporter-2
T2DM: type 2 diabetes mellitus
